# The Young Stethoscopist, or the Student's Aid to Auscultation

**Published:** 1846-07

**Authors:** 


The Young Stethoscopist, or the Student's Aid to Auscultation. By *Henry L. Bowditch,* M.D. 8vo. pp. 276. Boston, 1846. Ticknor and Co.

We have been much pleased on the whole with this manual, and can truly recommend it to the student of Auscultation as an excellent guide. We had hoped to have found space to have noticed its contents more at length; but the extension of some of the articles in the present number has put this out of our power. The very copious index adds very much to the value of the work. It would be well that authors in general would pay as much attention to this important point as Dr. Bowditch.

